# Farewell to Animal Testing: Innovations on Human Intestinal Microphysiological Systems

**DOI:** 10.3390/mi7070107

**Published:** 2016-06-27

**Authors:** Tae Hyun Kang, Hyun Jung Kim

**Affiliations:** Department of Biomedical Engineering, The University of Texas at Austin, Austin, TX 78712, USA; thkang@utexas.edu

**Keywords:** intestine, microphysiological system, gut-on-a-chip, microbiome, host-microbe interaction, inflammatory bowel disease, disease model

## Abstract

The human intestine is a dynamic organ where the complex host-microbe interactions that orchestrate intestinal homeostasis occur. Major contributing factors associated with intestinal health and diseases include metabolically-active gut microbiota, intestinal epithelium, immune components, and rhythmical bowel movement known as peristalsis. Human intestinal disease models have been developed; however, a considerable number of existing models often fail to reproducibly predict human intestinal pathophysiology in response to biological and chemical perturbations or clinical interventions. Intestinal organoid models have provided promising cytodifferentiation and regeneration, but the lack of luminal flow and physical bowel movements seriously hamper mimicking complex host-microbe crosstalk. Here, we discuss recent advances of human intestinal microphysiological systems, such as the biomimetic human “Gut-on-a-Chip” that can employ key intestinal components, such as villus epithelium, gut microbiota, and immune components under peristalsis-like motions and flow, to reconstitute the transmural 3D lumen-capillary tissue interface. By encompassing cutting-edge tools in microfluidics, tissue engineering, and clinical microbiology, gut-on-a-chip has been leveraged not only to recapitulate organ-level intestinal functions, but also emulate the pathophysiology of intestinal disorders, such as chronic inflammation. Finally, we provide potential perspectives of the next generation microphysiological systems as a personalized platform to validate the efficacy, safety, metabolism, and therapeutic responses of new drug compounds in the preclinical stage.

## 1. Introduction

### 1.1. Human Intestine, a Complex Organ to Mimic

The human intestine is a complex, but dynamic, organ where intestinal mucosa, commensal gut microbiota, and resident immune components, such as gut-associated lymphoid tissues (GALT), interact, stimulate, and respond to one another to maintain intestinal homeostasis [1]. Differentiated intestinal epithelium plays a pivotal role to present a physically tight junction barrier against foreign pathogens and an absorptive brush border to transport nutrients. The mucus layer provides a protective physical barrier preventing the invasion of pathogenic bacteria [2]. Commensal gut microbiota are colonized as multi-species communities in the intestinal lumen. All of these players always communicate with various antigen-presenting immune cells in the lamina propria, which is a key surveillance to maintain intestinal homeostasis and regulation [3]. Peristalsis is a form of mechanical deformation that induces proliferation and differentiation of intestinal epithelial cells [4]. More importantly, the rhythmical cyclic deformations may suppress the small intestinal bacterial overgrowth (SIBO) [5,6]. The balance of multi-species gut microbes during the host-microbe crosstalk shapes the immune milieu; likewise, mucosal immunity also influences the microbial signature [7]. Recent studies suggest that the perturbed population of gut microbiota (i.e., dysbiosis) often results in compromised intestinal functions and imbalanced subsets of key immune cells, which is one of the causative factors inducing chronic intestinal inflammation [8]. All of these complex crosstalks are tightly regulated by the structural, biological, chemical, physical, and physiological components in the gut microenvironment. Indeed, the majority of prevalent human gastrointestinal (GI) diseases, such as inflammatory bowel disease (IBD) or colorectal cancer (CRC), are closely associated with the compromised host-microbe crosstalks [9,10,11,12]. Hence, it is of great importance to have experimental models that can reproducibly reconstitute the aforementioned key elements in the mechanically-active microenvironment with three-dimensional (3D) tissue microarchitecture. However, due to its physiological complexity, development of a robust intestine model to study GI diseases and define new therapeutics remains a crucial unmet need.

### 1.2. Limitations in Animal Models

In the drug development process, animal models have been abundantly used to validate the efficacy and toxicity of new drug compounds in the preclinical stage [13,14,15,16]. Especially for the drugs targeting per-oral administration, multiple factors such as bioavailability [17], first-pass effect [18], efflux system [19], or the role of gut microbiota [20], can influence the efficiency and safety of the drug. Although animal tests provide a wide range of systematic responses as similar to the human physiology, these models are resource-intensive (time, labor, money, or amount of drug compounds), ethically questionable, and low-throughput [21,22]. More importantly, due to the species difference between animals or animal and humans [23,24], prediction of drug responses in human, such as pharmacokinetics (PK) and pharmacodynamics (PD), undergoes a huge challenge. Thus, discovering a new in vitro model of the human intestine that can reconstitute the 3D intestinal structure, recapitulate the biological and physical functions, and recreate the pathophysiological responses under disease-like perturbations is a critical unmet need.

Another key aspect of the new intestine model is to have modularity in the system to tune the different levels of complexity. To understand the pathophysiology of human intestine, it is required to dissect the etiological factors or interacting components independently. However, it has been extremely difficult to perform the independent control of disease-initiating factors in animal models. For instance, IBD, such as Crohn’s disease (CD) or ulcerative colitis (UC), is a complicated disease causing chronic intestinal inflammation [25,26]. Aberrant interactions between luminal microbiome and immune components in the lamina propria seem to cause IBD, but it is still unclear “who” triggers the heterogeneous pathologies in various IBD patients [11,12]. This is an important question because determining the triggering factor may define the therapeutic target for the IBD interventions. Since many intestinal diseases have similar complexities in terms of the initiation, development, and prognosis, modular capability of the model systems to independently manipulate the role of each component is a cogent feature to be accomplished in the non-animal intestine models. In the next section, we list existing non-animal human intestine models and discuss the advantages and potential drawbacks to understand human intestinal diseases.

## 2. In Vitro Models to Mimic Tissue-Level Intestinal Pathophysiology

### 2.1. Cell Culture Models (2D)

**Figure 1:**
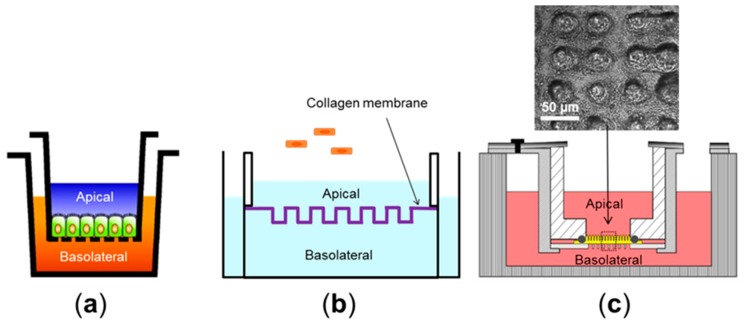
Schematics of static culture models using human intestinal epithelial cells: (**a**) an epithelial monolayer has been grown on a semipermeable insert in the Transwell; (**b**) a collagen membrane patterned with crypt-like topography is incorporated in replacement of the porous membrane in the Transwell [34]. Caco-2 cells (orange) can overlay the topological collagen surface to form a monolayer; and (**c**) a pseudo-3D intestine model has the hydrogel-based villous microarchitecture. An inset image shows a top view of Caco-2 cells grown on villous scaffolds. Reproduced by permission of John Wiley and Sons. All rights reserved [35].

Human colorectal adenocarcinoma lines such as the Caco-2 (absorptive, enterocyte-like) or HT-29 (mucus-secretory, goblet-like) have been used as a source of human intestinal epithelial cells. They have been abundantly used for intestinal permeability assays in vitro using the porous membrane-equipped static culture system (e.g., Transwell) [27]. Transwell culture is a simple and robust system to form a tight junction barrier of an intestinal epithelial monolayer. A polarized monolayer grown on a semi-permeable nanoporous membrane provides apical (luminal) and basolateral (abluminal) compartments (Figure 1a) [28], where bacterial cells or leukocytes can be transiently added into each side, respectively, to induce inflammatory immune responses. For instance, peripheral blood mononuclear cells (PBMC) or isolated tissue macrophages can be introduced into the basolateral side after Caco-2 cells form a monolayer [29]. Under this condition, epithelial cells produce considerable amount of proinflammatory cytokines, such as tumor necrosis factor (TNF)-α and interleukin (IL)-1β in the presence of both bacteria and PMBC in the apical and basolateral side, respectively [29]. This observation recapitulates the intestinal inflammatory responses elicited in vivo during the host-microbe crosstalks. Cell culture models can also be used to validate the toxicology studies of nanoparticles on intestinal iron adsorption. Exposure of high dosage (2 × 10^11^ particles/mL for 50 nm particles and 1.25 × 10^12^ particles/mL for 200 nm particles) of polystyrene nanoparticles on the apical side increased levels of iron transport through a Caco-2 monolayer compared to the unexposed control. This result is consistent with the results of an in vivo chicken intestine model where the chronic exposure of the nanoparticles (2 mg/kg) resulted in remodeling of intestinal villi and damaged epithelium [30].

Despite the versatile advantages of the cell and tissue cultures, static cultures of intestinal epithelial cells in the Transwell format often fail to induce in vivo-like 3D villous morphology [31,32]. A lack of the 3D microenvironment of the small intestine may lead to false results due to the poor cytodifferentiation [33]. A lack of mechanical deformations, as well as fluid shear stress, are other key limitations in the static culture models [31].

### 2.2. Cell Culture Models (Pseudo-3D)

While Transwell cultures have predominantly presented 2D cell growth with a monolayer, recent in vitro culture techniques have significantly improved from 2D to 3D [33]. Caco-2 cells are able to grow crypt-like topography on a polydimethylsiloxane (PDMS) surface coated with fibronectin, in which cells exhibit similar physiological functions, such as alkaline phosphatase expression and mitochondria activities, to the levels observed in human intestinal crypts [36]. This crypt-mimicking culture also results in decreased barrier functions measured by transepithelial electrical resistance (TEER) values than the 2D flat substrate, supporting the in vivo relevance of the barrier integrity of crypt cells (Figure 1b) [34]. Three-dimensional scaffolding of villus microstructure using hydrogel can mimic the finger-like projections covered by epithelial cells [37] (Figure 1c). These pseudo-3D villi lined by a monolayer of gut epithelium on hydrogel promote drug adsorption (e.g., antenolol) with a strong correlation with human intestines than conventional 2D culture models [35]. On the other hand, this method does not provide capillary components. Moreover, a monolayer of Caco-2 cells without villous cytodifferentiation is questionable to demonstrate adequate physiological functions of basal crypts. Alternatively, it has been possible to co-culture intestinal enterocytes (e.g., Caco-2) with mucus-producing epithelial cells (e.g., HT29-MTX) [38]. However, this method can potentially mislead to imbalanced populations of absorptive and goblet cells because the growth rate of the HT29-MTX line is significantly faster than that of Caco-2, by which the initial seeding ratio of 9:1 (absorptive:goblet) is rapidly compromised over time, resulting in non-physiological artifacts in terms of the cell populations.

### 2.3. Organoid Culture Models (3D)

Advances in cell sorting technology have capacitated to isolate leucine-rich repeat-containing G-protein coupled receptor 5 positive (LGR5+) small intestinal stem cells, by which the use of LGR5 cells provides the formation of 3D intestinal organoids with crypt–villus characteristics [39]. For maintaining the functional crypt-villus characteristics for a long-term period, stem cell niches should be sustained in the 3D hydrogel with key soluble factors, such as epidermal growth factor (EGF), Wnt3A, R-spondin, and Noggin [40,41,42]. Direct differentiation of human embryonic and induced pluripotent stem cells (iPSCs) has also enabled the recreation of complex 3D intestinal organoids [43]. Crypt samples isolated from individual patients can provide patient-specific organoids, which may allow personalized therapeutics [44]. The organoids can be further differentiated into more a physiologically-relevant structure by introducing their supporting mesenchymal cells [45]. Intestinal organoids have been utilized to demonstrate *Clostridium difficile* (*C. diff*) infection on the lumen side of organoids. The *C. diff* infection increases paracellular permeability [46], and reduces Na^+^/H^+^ exchanger 3 (NHE3) and mucin 2 (MUC2) expression to enhance colonization [47,48]. Intestinal stem cells isolated from the basal crypts have also been cultured to generate “enteroids” or “colonoids” [49]. Human enteroids have been used to demonstrate infections of rotavirus [50] and norovirus [51], or the exposure to cholera toxin [52] or to enterohemorrhagic *Escherichia coli* (EHEC) [53]. The EHEC infection study reveals that the EHEC serine protease (EspP) resulted in redistribution of F-actin in both apical and basolateral membranes of enteroids, which causes the destruction of lateral intracellular interaction [54]. Although organoid culture models have suggested strong in vivo relevance with the cytodifferentiation and morphogenesis of the 3D crypt-villus axis, a lack of mechanical deformation and fluid shear stress are crucial drawbacks to sustain a stable host-microbe ecosystem. Furthermore, most of existing organoids have an enclosed lumen inside the core, whereas the crypt region is exposed to the other. Hence, it has been extremely difficult to induce appropriate luminal stimulation (e.g., administration of microbiome, food, drug, or toxin).

### 2.4. Microfluidic Culture Models

**Figure 2:**
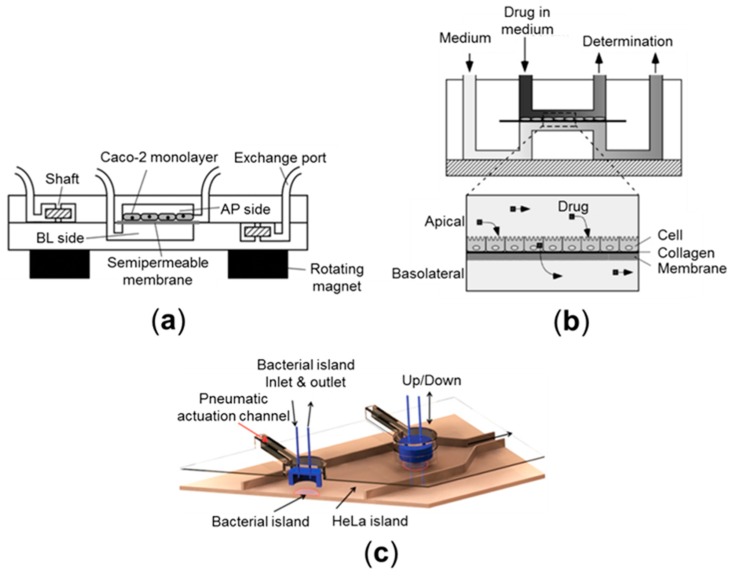
Schematic illustrations of microfluidic models mimicking human intestinal pathophysiology: (**a**) a microfluidic device consists of upper (AP side) and lower (BL side) layers integrated with stirrer-based micropumps and optical fibers. Caco-2 cells are cultured on a semipermeable membrane in the AP side culture chamber. Reproduced by permission of the Royal Society of Chemistry. All rights reserved [57]; (**b**) a cross-section view of a microfluidic device to evaluate intestinal absorption under fluidic conditions. Reproduced by permission of The Japan Society for Analytical Chemistry. All rights reserved [58]; and (**c**) a microfluidic model for the co-culture of epithelial and bacterial cells. The device provides pneumatically-actuated trapping regions for providing bacterial islands around epithelial cells. Each bacterial island (1200 mm in diameter with 1000 mm in distance) has a separate inlet and an outlet for providing nutrients and removing wastes from the island. Reproduced by permission of Royal Society of Chemistry. All rights reserved [62].

Microfluidic culture methods have enabled the introduction of direct fluid shear stress in a physiological range, by which the cells grown in a microfluidic channel take advantage of physical cues to initiate the physiological cytodifferentiation [55]. In addition, microfluidic approaches can provide a precise manipulation of patterned microchannels created by the photolithography method, by which flow direction, volumetric flow rate, shear stress, or chemical gradients can be tuned in a versatile way [56]. For instance, a microfluidic device combined with integrated micropumps and optical fiber sensors also enables long-term perfusion culture of intestinal epithelium, where the transport of rhodamine 123, a chemical dye to demonstrate the transport from the basolateral to the apical side (BL-to-AP), is monitored successfully (Figure 2a) [57]. Another study exhibits significant intestinal absorption of cyclophosphamide, a chemotherapeutic compound, using a microfluidic device containing a semipermeable membrane lined by a Caco-2 monolayer under the controlled fluid shear consistent with physiological condition (Figure 2b) [58]. A microscale cell culture analog of the GI tract demonstrates the metabolism of acetaminophen by passing it through the systemic circulation, then subsequently delivering it to the liver cells to measure liver toxicity in a dose-dependent manner. Results in this study are consistent with in vivo metabolism in mice, suggesting the promising perspective of the system to toxicology studies for orally-administered drugs [59]. A microfluidic culture integrated with a micro-porous membrane covered by finger-like silicon shapes creates a 3D microarchitecture similar to intestinal villi and a tight junction [60]. This microfluidic device enhances barrier function (quantitated by TEER) of cultured Caco-2 cells up to four-fold higher than the results in static Transwell cultures [31], suggesting that the perfusion flow at a low shear stress recreates a better tight junction barrier. However, this model still lacks mechanical deformations that are always present in a normal in vivo intestine, which hampers the demonstration not only of drug absorption and toxicity, but also intestinal homeostasis [61].

## 3. Human Gut-on-a-Chip: Emulating Organ-Level Intestinal Pathophysiology

Aforementioned experimental approaches that recapitulated the feature of the living human intestine have demonstrated, in part, the validity of drug screening in a reductionist approach. To study human intestinal diseases there are two major challenges that should be contemplated. First, some of the models that we discussed have a 2D morphological nature and static culture condition. Thus, the lack of physiological 3D miniaturization and physiological culture methods are serious drawbacks. In addition, limited physiological reconstitution may hamper replaying appropriate key disease factors in the system. For instance, if a model does not reproduce mechanical deformations, it never recreates the mechanical deformation-associated diseases, such as ileus. In addition, although conventional cell culture models may provide a precise subset of experiments to dictate pharmacodynamics of potential therapeutics or interventions (e.g., drug treatment to inflamed cells cultured on the Transwell), understanding how to reproducibly demonstrate and predict the pharmacokinetics of specific therapeutics in a model system remains a critical concern. Thus, we further discuss how these questions, in terms of the involvement of intestinal key components, have been addressed and improved.

### 3.1. Peristalsis

**Figure 3:**
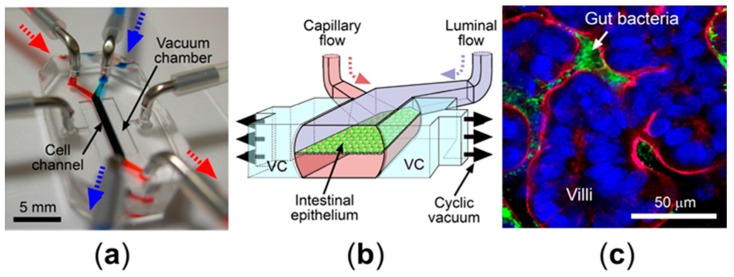
An organomimetic human gut-on-a-chip microphysiological system: (**a**) a photographic image of a gut-on-a-chip microdevice. Arrows indicate the direction of flow in the microchannels (blue, luminal flow; red, capillary flow); (**b**) a schematic of a gut-on-a-chip displaying the flexible nature to exert peristalsis-like, vacuum-driven mechanical deformations. VC, vacuum chamber; and (**c**) an overlaid confocal immunofluorescence image showing a horizontal cross-section view of intestinal villi co-cultured with GFP *E. coli* (green). Brush border membrane (F-actin, red) and nuclei (DAPI, blue) are fluorescently highlighted.

Bowel movements, such as macroscopic peristalsis, as well as microscopic villus motility, primarily promote the proliferation and differentiation of epithelial cells in the intestine [4]. Interestingly, mechanical deformations can restrict the aberrant overgrowth of microbial cells in the intestinal lumen, known as SIBO [5]. Thus, it is of great importance to employ peristalsis-like mechanical deformations in human intestinal disease models. A cell-stretching pneumatic module has been devised to exert stretching motions, by which the intensity and frequency of cell strains are programmable [63,64]. The polymeric cell stretching modules have been employed in a biomimetic human “Gut-on-a-Chip” microphysiological system [6,31,32,65,66] to introduce cyclic mechanical distortions on a microfluidic device (Figure 3a). The gut-on-a-chip has two juxtaposed parallel microchannels separated by a porous, extracellular matrix (ECM)-coated flexible membrane made by PDMS. Beside the cell microchannels, there are two apposed hollow vacuum chambers connected to the computer-mediated vacuum controller, by which the cyclic rhythmical motions can be induced by the repeated vacuum applications (Figure 3b). As a result, intestinal epithelial cells grown on the central cell microchannel can undergo physical deformations with the same elongation intensity and frequency applied to the vacuum chamber. In this condition, Caco-2 cells spontaneously undergo villus morphogenesis followed by the cytodifferentiation with four lineages of differentiated small intestinal epithelial cells (absorptive, mucus-secretory, enteroendocrine, and Paneth) [32]. It has been known that the paracellular permeability in the Caco-2 monolayer in the static culture is much lower than in vivo, owing to the unstirred water layer, which makes an artifact permeability in vitro compared to results observed in the system with dynamic shear stress and peristalsis-like motility [67]. Importantly, this peristalsis-like motion significantly enhances paracellular permeability of Caco-2 cells in combination with fluid flow, while the presence of microfluidic flow alone does not significantly change the permeability [31]. In this experimental setup, cyclic motions do not change the TEER level, indicating that the dynamic peristalsis-like deformations and trickling flow in vitro significantly enhance the paracellular transport without compromising the tight junction barrier.

Mechanical deformation that contributes to the peristalsis is also important to maintain the controlled population of microbiota in the gut. For instance, impaired bowel movements in patients of SIBO or postoperative ileus often result in the dysbiosis or overgrowth of gut microbiota [68]. The gut-on-a-chip microphysiological system also reveals the contribution of peristalsis on SIBO, where the mechanical deformation alone is sufficient to regulate the overgrowth of lab strain *E. coli* in the microengineered villi [6]. This is an important discovery because there have been no experimental models to employ both fluid shear stress and mechanical deformations. In this report, the gut-on-a-chip successfully decouples both physical factors, then assesses the effect of mechanical tensions. This result also strengthens the hypothesis that the epithelial distortion inhibits bacterial colonization [5].

### 3.2. Host-Microbe Ecosystem

**Figure 4:**
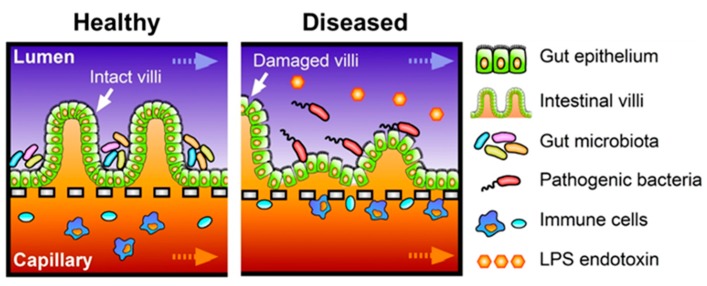
A simplified schematic illustration comparing the human intestinal microenvironment in healthy versus diseased conditions caused by chronic inflammation. In the diseased condition, inflamed intestinal microenvironment results in the leaky gut epithelium in accordance with the infection of pathogenic bacteria, increased level of bacterial endotoxins such as lipopolysaccharides (LPS), and recruitment of circulating immune cells.

In general, maintaining the stable balance between gut microbiota and host cells, including immune components, is directly related to the homeostasis of intestine [1]. In other words, dysbiosis of microbiota or the imbalance of crosstalk between microbiota and host cells may increase the chance of intestinal diseases such as IBD [12]. Hence, demonstration of a stable host-microbe ecosystem is another important physiological feature of human intestinal disease models. However, existing static culture models cannot maintain a certain population of bacterial cells because bacteria always outgrow. Alternatively, microfluidic culture can be considered to maintain regulated numbers of microbial cells in a system. For example, microfluidic co-culture of HeLa cells and commensal *E. coli* is enabled by a pneumatically-actuated system to form biofilm, followed by introducing EHEC, where the commensal biofilm protects EHEC infection (Figure 2c) [62]. However, this report demonstrated the segregated compartment between host HeLa cells and microbial cells by a pneumatically-controllable PDMS wall, whereas direct contact between host and microbial cells is abundant in vivo. More advanced host-microbe co-cultures have been accomplished in the human gut-on-a-chip, where Caco-2 cells grown into 3D villi stably maintained their viability and functionality in the presence of *Lactobacillus rhamnosus* GG (LGG) cells [31] or an over-the-counter probiotic formulation with eight different gut bacteria, VSL#3 [6]. Of great interests, transcriptome profiles of Caco-2 cells grown into villi in the presence of VSL#3 microbial cells show the highest similarity with the gene expression profiles in the ileum obtained from healthy human individuals, whereas the transcriptomes of Caco-2 cells grown in the static Transwell show significantly different patterns from either of them [6]. Furthermore, when non-pathogenic laboratory strains of *E. coli* bacteria are added to the luminal side of the system, they start to colonize in the intervillus space, but failed to decrease the barrier function. On the contrary, when pathogenic enteroinvasive *E. coli* (EIEC; serotype O124:NM) are introduced in the lumen mimicking pathogenic infection, they overgrow in the luminal region of the villi within 24 h, and rapidly disrupt the villus morphology and shorten the height of villi. Most importantly, the gut-on-a-chip microsystem can demonstrate the probiotic therapeutics as a proof-of-principle, where the pre-colonization of VSL#3 cells prior to the EIEC infection significantly suppresses the overgrowth of EIEC bacteria. Moreover, VSL#3 cells significantly delay the intestinal injury caused by the vigorous interactions between EIEC and PBMCs, supporting the replication of probiotic interventions to IBD patients [69,70,71]. As a future direction, gut-on-a-chip can be utilized to investigate host-pathogen interactions, such as the recurrent infections of *C. diff* in IBD patients [72] or compromised mucosal permeability of the stomach and the intestine after *Helicobacter pylori* infection [73] using the gut-on-a-chip co-culture system. The human gut-on-a-chip device is indeed the first in vitro model that both intestinal cells and commensal bacteria could be co-cultured over a week or longer under regulated flow rate and mechanical stretching motions that allows intestinal cells to be differentiated properly without detectable bacterial overgrowth, which is more physiologically relevant to better mimic pathophysiology of intestinal diseases on-chip (Figure 4).

### 3.3. Immune Components

Immune components are another crucial feature that should be considered in the intestinal disease models, such as IBD or CRC. Since PBMCs contain multiple populations of white blood cells, and the protocol to obtain PBMCs is relatively simple by taking a “buffy coat” through multiple centrifugations, PBMCs have been most abundantly used for the epithelium-immune or bacteria-immune interactions [74]. For instance, PBMCs used in intestinal inflammation models include mixed populations of innate (monocytes, natural killer cells) and adaptive (B and T cells) immune cells [29,75]. In a similar approach, PBMCs are also used in a recent inflammation model performed by the gut-on-a-chip, where the crosstalk between LPS or non-pathogenic *E. coli* in the lumen side and PBMCs in the capillary side results in the directional secretion of proinflammatory cytokines including IL-1β, IL-6, IL-8, and TNF-α significantly [6]. In addition, villus epithelium challenged by both LPS and PBMCs significantly upregulates 36 genes that are associated with early inflammatory signaling. PBMCs applied in the capillary side also interact with non-pathogenic or pathogenic *E. coli* strains [6]. A bottom line of these experiments is that the absence of immune components, LPS, or non-pathogenic *E. coli* challenges does not induce a significant level of inflammation, suggesting that the incorporation of immune components on the system is essential for the intestinal disease models.

## 4. Prospects

The intestine is a dynamic organ that includes highly complex tissue-specific functions (e.g., host-gut microbiota interactions [76]), as well as inter-organ interactions (e.g., gut-liver axis [77], gut-brain axis [78]). As mentioned before, the balance among microbiota or between microbiota and the host immune system is of great importance to maintain intestinal homeostasis; in other words, losing the balance preferably causes intestinal diseases. Human intestinal microphysiological systems have suggested a new avenue to rebuild predictive disease models, test targeted therapeutics, validate previously existing therapeutics, or dissect complex disease mechanisms. In this Prospects section, we focus on the new territory that previous model systems have not sufficiently discovered.

### 4.1. Pharmaceutical Applications

Pharmaceutical companies have heavily relied on the in vivo animal models to validate PK information including absorption, distribution, metabolism, and excretion (ADME) and PD, as well as toxicity and safety in the preclinical stage. However, animal models occasionally fail to precisely predict the human responses mainly because of the species discrepancies with humans in terms of drug metabolism, transport, or efflux system in the GI tract. On the contrary, in vitro models can utilize human cell-based culture protocols, by which primary or intestinal stem cells can be considered to maximize the in vivo relevance. Since the enterocytes in the intestine show strong drug metabolism with high cytochrome P450 enzymatic activities, reliable intestine models for pharmaceutical applications should provide key drug metabolizing functions, per se. Alternatively, in vitro models have been used to co-culture Caco-2 cells, HepG2/C3a liver cells, and human breast carcinoma MCF-7 cells in separate compartments, which have then been connected through microfluidic channels to validate intestinal absorption, hepatic metabolism, and bioactivity of anticancer agents, such as cyclophosphamide and epirubicin [79,80]. The microfluidic platform using multiple Transwells is also proposed to predict human intestinal and hepatic metabolism of paracetamol clearance and bioavailability, respectively [81]. An openable artificial micro-tube device containing Caco-2 cells is another possible consideration in terms of in vitro small intestine mimicry that is used for ADME of hydrophilic and hydrophobic drugs [82]. However, a lack of mechanical bowel movements and host-microbe co-culture is a major concern of those systems to be improved.

**Figure 5:**
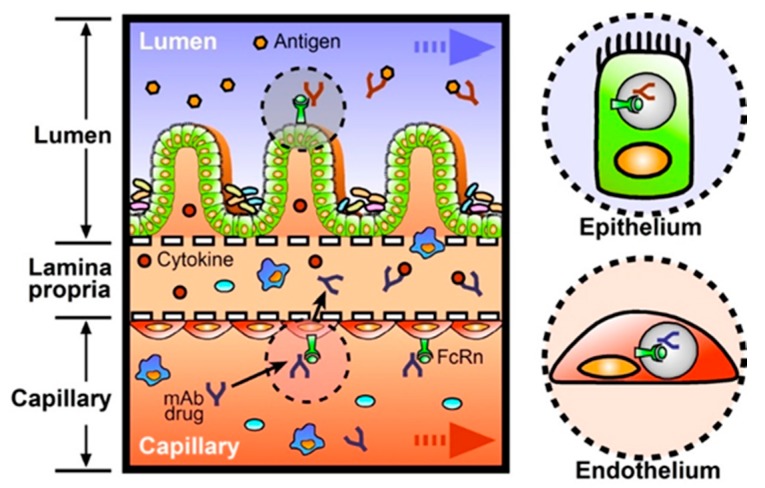
A schematic diagram of the transcytosis of monoclonal antibody (mAb) drugs across the lumen-mesenchyme-capillary tissue interface. Intravenously-administered mAb drugs (blue) bind to the neonatal Fc receptor (FcRn), in which the internalized endosome functions as a vehicle to carry this complex from the capillary layer into the lamina propria (a right bottom inset with a dotted circle). Released mAb drugs capture disposed proinflammatory cytokines (secreted by activated immune cells during IBD. Orally-delivered mAb drugs (red) can also be tested in this microphysiological system in situ (a right top inset with a dotted circle).

There have been increasing numbers of new FDA-approved drugs leveraging monoclonal antibodies (mAb) for targeted medication [83]. For instance, IBD treatment with mAb that suppress aberrant immune responses in the intestine has changed the clinical management of IBD with a few significant advantages of high specificity, less immunogenicity, and reduced toxicity [84]. The mAb drugs in clinics target proinflammatory cytokines, such as TNF-α, to block their unwanted immune-activating function (Table 1). These cytokines are produced by the resident immune cells recruited to the lamina propria during gut injury or inflammation [85]. To effectively function as anti-inflammatory drugs, therapeutic antibodies should be transported from vascular or lymphatic capillaries to lamina propria to capture the cytokines (Figure 5). Thus, it is important to investigate how much mAb can be transported through the vascular endothelium, as most of the therapeutic antibodies are injected intravenously. Neonatal Fc receptor (FcRn) is a major contributor to enhance the transcytosis of immunoglobulin G (IgG) antibody and increase serum half-life in humans [86]. Normally the human FcRn (hFcRn) is expressed on both endothelial and intestinal epithelial cells in vivo [87,88]. In addition, human intestinal cell lines including Caco-2 cells also express hFcRn [89,90], suggesting that IgG antibodies can be transcytosed from the capillary side to the lumen side. This proposed transport event of IgG antibodies occurs from the basolateral membrane to the intestinal lumen in the hFcRn-transgenic mice model [91]. However, this event does not arise in wild-type mice because FcRn is not highly expressed in the intestine of adult rodents [92,93], suggesting that the expression level of FcRn is critical for the proper delivery of mAb drugs. Thus, we envision the value of a human organ mimicry model to predict the transport profile of IgG antibody drugs through the human intestinal epithelial layer. The human gut-on-a-chip, or any equivalent in vitro intestine models, are highly appreciated to test the effective delivery, transport, function, and excretion of mAb drugs in the presence of physical bowel movement, gut microbiome, and immune components. For example, the cyclic mechanical strain may contribute to affect the extent of hFcRn-mediated transcytosis of IgG antibodies, just as we pointed out for the general transcytosis events in vivo. Furthermore, gut-on-a-chip may be exploited to validate orally-administered mAg drugs with apposite formulations, where the transcytosis of mAb drugs from the gut lumen to lamina propria can be validated on-chip (Figure 5).

### 4.2. Applications in the Food Industry

We also anticipate the contribution of human intestinal microphysiological systems on the nutraceutical science and food industry. For instance, non-caloric artificial sweeteners (NAS) have been used worldwide to substitute sugar (sucrose). While the use of NAS is recognized as safe and beneficial due to their low caloric contents, recent studies suggest that the use of NAS may not be fully appreciated because it can cause dysbiosis of gut microbiota or metabolic abnormality [94]. To further investigate this finding, mice models can be primarily considered along the line of their gut microbiota. However, although humans and mice share some intestinal commensal bacteria, such as *Firmicutes*, *Bacteroidetes*, and *Proteobacteria* phyla [95], discrepancies in the microbial signature between human and mice is not negligible. Therefore, it is necessary to assess how the intake of NAS changes the human intestinal host-microbiota ecosystem and intestinal metabolism. Hence, human relevant microphysiological gut models should be considered, and the validation of NAS using gut-on-a-chip microsystems may be a plausible proof-of-principle study for the expanded applications in other food components.

### 4.3. Revalidation of Probiotics and Prebiotics

Another possible application is the revalidation of pro- and prebiotics effects as a food supplement [96]. Prebiotics generally indicate the non-digestible fibers that cannot be cleaved by human intestinal digestive enzymes, whereas the majority of gut microbiota can utilize the prebiotic fibers as energy sources. Some commensal microbes have enzymes to degrade prebiotic polymers, by which subsequent microbial populations perform degradation or fermentation to produce major microbial metabolites, such as short-chain fatty acids (SCFA). Since the SCFAs play a key role to support energy sources to the enterocytes, as well as to orchestrate the homeostasis of mucosal immune system [97], defining the prebiotic therapy in combination with probiotic health-promoting microbiota (i.e., synbiotics [98]) is prodigious in GI clinics, as well as nutraceutical dairy product industries.

## 5. Conclusions

The physiological legitimacy of the human-relevant gut models includes fully-differentiated intestinal epithelial cells, crypt-villus 3D microarchitecture, metabolically active gut microbiota, functional immune components, and a mechanically dynamic microenvironment [66]. Biomimetic or biologically-inspired engineering approaches enable the reconstitution of the minimal set of structural and functional units, where the disease etiological factors can be considered to recapitulate the disease-specific pathophysiology. Since the microphysiological system is capable of independently manipulating physiological aspects in a modular way, this approach provides a better platform as a model system to scrutinize which factor contributes to the maintenance of human intestinal physiology or the development of diseases. We believe current microphysiological systems will be further improved by incorporating human intestinal stem or iPS cells and patient samples through the intensive collaborations mainly between biomedical engineers and GI clinicians. More clinical and biomedical inputs will lead the evolution of intestinal microphysiological systems, by which the first generation of these microsystems, including gut-on-a-chip, will transform into the second generation with concepts of human “Disease-on-a-chip” or “Patient-on-a-chip” to target personalized, patient-oriented, customized diagnostics and therapeutics. Ultimately, we expect that the human intestinal microphysiological systems can contribute to the Precision Medicine Initiative [99] as a trendsetter in the near future. Taken together, progress in human intestinal organs-on-chips microsystems may accelerate the drug development process and replace animal testing.

## Figures and Tables

**Table 1:** List of therapeutic monoclonal antibodies used for IBD interventions.

Name	Trade Name	Application	Target	Type
Adalimumab	Humira	CD	TNF-α	Mab ^1^
Certolizumab pegol	Cimzia	CD	TNF-α	Fab' ^2^
Fontolizumab	HuZAF	CD	IFN-γ	Mab
Infliximab	Remicade	CD/UC	TNF-α	Mab
Natalizumab	Tysabri	CD	integrin α_4_	Mab
Visilizumab	Nuvion	CD/UC	CD3	Mab

Note: ^1^ full-length monoclonal antibody; ^2^ fragment, antigen-binding, including hinge region (one arm).

## Abbreviations

The following abbreviations are used in this manuscript:

2D: Two-dimensional
3D: Three-dimensional
CD: Crohn’s disease
CRC: Colorectal cancer
DAPI: 4',6-diamidino-2-phenylindole
ECM: Extracellular matrix
EHEC: Enterohemorrhagic *E. coli*
EspP: EHEC serine protease
FcRn: Neonatal Fc receptor
GFP: Green-fluorescence-labeled protein
GALT: Gut-associated lymphoid tissues
IBD: Inflammatory bowel disease
IgG: Immunoglobulin G
IL: Interleukin
iPSCs: Induced pluripotent stem cells
LGG: *Lactobacillus rhamnosus* GG
LGR5: Leucine-rich repeat-containing G-protein coupled receptor 5
LPS: Lipopolysaccharide
MUC2: Mucin 2
NAS: Non-caloric artificial sweeteners
NHE3: Na^+^/H^+^ exchanger 3
PBMCs: Peripheral blood mononuclear cells
PD: Pharmacodynamics
PDMS: Polydimethylsiloxane
PK: Pharmacokinetics
SCFA: Short-chain fatty acids
SIBO: Small intestinal bacterial overgrowth
TEER: Trans-epithelial electrical resistance
TNF: Tumor necrosis factor
UC: Ulcerative colitis
